# Hyperkinetic stereotyped movements in a boy with biallelic CNTNAP2 variants

**DOI:** 10.1186/s13052-021-01162-w

**Published:** 2021-10-12

**Authors:** Marcello Scala, Midas Anijs, Roberta Battini, Francesca Madia, Valeria Capra, Paolo Scudieri, Alberto Verrotti, Federico Zara, Carlo Minetti, Sonja C. Vernes, Pasquale Striano

**Affiliations:** 1 Pediatric Neurology and Muscular Diseases Unit, IRCCS Istituto Giannina Gaslini, Genoa, Italy; 2 Department of Neurosciences, Rehabilitation, Ophthalmology, Genetics, Maternal and Child Health University of Genoa, Genoa, Italy; 3 Neurogenetics of Vocal Communication Group, Max Planck Institute for Psycholinguistics, Nijmegen, The Netherlands; 4 Department of Clinical and Experimental Medicine, University of Pisa, Pisa, Italy; 5 IRCCS Fondazione Stella Maris, Pisa, Italy; 6 Medical Genetic Unit, IRCSS Istituto G. Gaslini, Genoa, Italy; 7 Department of Pediatrics, University of Perugia, Perugia, Italy; 8 School of Biology, University of St Andrews, Fife, UK; 9 Donders Institute for Brain, Cognition and Behaviour, Nijmegen, The Netherlands

**Keywords:** CASPR2, Intragenic duplication, Hyperkinetic movement disorder, Hyperkinesia, Speech impairment, Intellectual disability, Autism

## Abstract

**Background:**

Heterozygous variants in *CNTNAP2* have been implicated in a wide range of neurological phenotypes, including intellectual disability (ID), epilepsy, autistic spectrum disorder (ASD), and impaired language. However, heterozygous variants can also be found in unaffected individuals. Biallelic *CNTNAP2* variants are rarer and cause a well-defined genetic syndrome known as CASPR2 deficiency disorder, a condition characterised by ID, early-onset refractory epilepsy, language impairment, and autistic features.

**Case-report:**

A 7-year-old boy presented with hyperkinetic stereotyped movements that started during early infancy and persisted over childhood. Abnormal movements consisted of rhythmic and repetitive shaking of the four limbs, with evident stereotypic features. Additional clinical features included ID, attention deficit-hyperactivity disorder (ADHD), ASD, and speech impairment, consistent with CASPR2 deficiency disorder. Whole-genome array comparative genomic hybridization detected a maternally inherited 0.402 Mb duplication, which involved intron 1, exon 2, and intron 2 of *CNTNAP2* (c.97 +?_209-?dup). The affected region in intron 1 contains a binding site for the transcription factor FOXP2, potentially leading to abnormal *CNTNAP2* expression regulation. Sanger sequencing of the coding region of *CNTNAP2* also identified a paternally-inherited missense variant c.2752C > T, p.(Leu918Phe).

**Conclusion:**

This case expands the molecular and phenotypic spectrum of CASPR2 deficiency disorder, suggesting that Hyperkinetic stereotyped movements may be a rare, yet significant, clinical feature of this complex neurological disorder. Furthermore, the identification of an in-frame, largely non-coding duplication in *CNTNAP2* points to a sophisticated underlying molecular mechanism, likely involving impaired FOXP2 binding.

**Supplementary Information:**

The online version contains supplementary material available at 10.1186/s13052-021-01162-w.

## Background

Biallelic *CNTNAP2* variants cause CASPR2 deficiency disorder (CDD), a syndromic neurodevelopmental disorder involving refractory epilepsy, ID, language impairment, and autistic features [1–4]. Heterozygous variants in *CNTNAP2* have also been identified in patients with a wide range of complex neurological phenotypes, but such variants can also be found in unaffected individuals [5, 6].

CASPR2, the protein product of *CNTNAP2*, is a transmembrane cell adhesion molecule from the neurexin family that is widely expressed throughout the brain [5, 7]. CASPR2 localises to juxtaparanodes of myelinated axons, where it is involved in neuron-glia interactions, and mediates the clustering of potassium channels via interaction with contactin 2 (also known as TAG-1) [8, 9]. CASPR2 is also localised to the synapse where it is involved in several additional processes, such as neuronal migration, neurite development and synapse maturation, stability, and function [10–14].

We describe a patient carrying compound heterozygous variants in *CNTNAP2* including a missense variant and an intragenic duplication that were inherited from the father and mother, respectively. In addition to the common features found in CASPR2 deficiency disorder (ID, ADHD, ASD, and speech impairment), the boy presented with peculiar hyperkinetic stereotyped movements, expanding the molecular and phenotypic spectrum of CDD.

## Case presentation

**Fig. 1:**
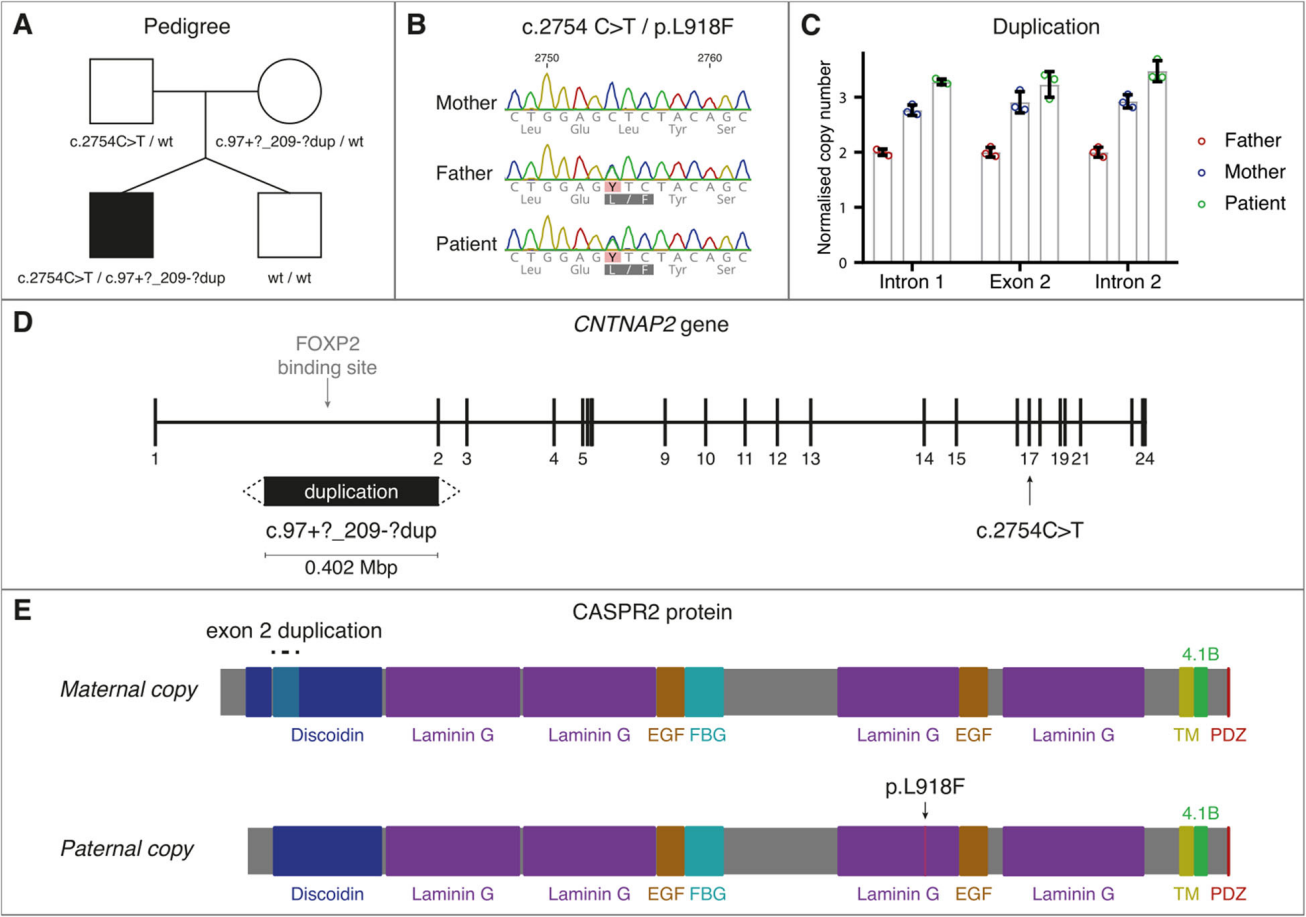
Genetic findings in the reported patient. (**A**) Pedigree of the family showing the affected patient carrying compound heterozygous variants in *CNTNAP2*: the paternally inherited missense c.2752C > T, p.(Leu918Phe) (ENST00000361727.3) in exon 17 and the maternally inherited c.97 +?_209-?dup duplication in intron 1 (for which the exact breakpoints have not been mapped). (**B**) Sanger sequencing traces showing the heterozygous single nucleotide variant in the proband and his father, consisting of leucine to phenylalanine substitution. (**C**) Confirmation of exon 2 duplication. RT-qPCR from genomic DNA shows that the patient and his mother have one extra copy of exon 2 as compared to the father. The duplication breakpoint is located after exon 2 since the first ~ 200 bp of intron 2 are still duplicated. Three technical replicates per condition. (**D**) Localization of inherited mutations on *CNTNAP2*. The maternally inherited duplication begins in the first intron and overlaps a binding site for the transcription factor FOXP2. Approximate boundaries of the duplication could be determined from the array-CGH and PCRs (see also supp material), indicating that the duplication also involves exon 2 and part of intron 2. The paternally inherited mutation is located in exon 17. (**E**) Predicted consequences of *CNTNAP2* variants at the protein level. Exon 2 encodes the first part of the discoidin domain, the sequence of which is duplicated by the maternally inherited c.97 +?_209-?dup variant. The paternally inherited p.(Leu918Phe) variant results in a amino acid change in exon 17, which encodes the third laminin G domain of CASPR2. Both domains belong to the extracellular region of CASPR2 which facilitates protein-protein interactions. Supplementary data contain additional details on the methodology and results. Discoidin = Discoidin homology domain, Laminin G = Laminin G domain, EGF = EGF-like domain, FBG = Fibrinogen-like region, TM = transmembrane domain, 4.1B = Protein 4.1 binding domain, PDZ = PDZ interaction domain


This case was a 7-year-old boy without a family history of neurodevelopmental disability, born at term to nonconsanguineous healthy parents (Fig. 1A) following a twin pregnancy complicated by intrauterine growth restriction and preeclampsia. His dizygotic twin brother was healthy at birth but was diagnosed with absence epilepsy during infancy. The neonatal course was characterised by feeding difficulties leading to failure to thrive. At 3 months of age, recurrent episodes of crying associated with semi-continuous, repetitive jerky movements of upper and lower limbs were observed, which were diagnosed as hyperkinetic stereotyped movements (Supplementary Video 1).

**Additional file 1.**


These paroxysmal hyperkinetic-dyskinetic episodes recurred periodically without any trigger, lasted 10–12 h, and mimicked infantile colics. Their frequency was temporarily reduced when anti-reflux formulas and ranitidine were administered for concomitant recurrent vomiting. Electroencephalogram and brain magnetic resonance imaging were unremarkable.

During his first years of life, the patient displayed ADHD and autistic traits. The boy also featured a phonological processing deficiency and clumsiness in gross and fine movements was also observed. His neuropsychological evaluation (WPPSI-III at age 6 years) revealed a global IQ of 71 (verbal, 86; performance, 68; processing, 55). The movement disorder persisted during the following years through non-triggered fast, high-amplitude, rhythmic, continuous and repetitive shaking involving the four limbs with stereotypic features (Supplementary Videos 2–3). His stereotypic movement disorder had been misdiagnosed as tics before, which indeed tend to appear from age 6–7 years [15]. However, these repetitive movements already started before the age of 3 and consisted of intense patterns of movement that ran longer than tics and were more bilateral than tics. Several medications, including carbamazepine, valproate, gabapentin, levodopa, flunarizine, benzodiazepines, did not improve his symptoms. The boy is currently receiving clonazepam, 0.2 mg/Kg in two daily doses.


**Additional file 2.**


### Genetic findings

Written informed consent was obtained from the patient’s parents. Whole-genome array-CGH on peripheral blood genomic DNA of the family quartet revealed a maternally inherited *CNTNAP2* variant c.97 +?_209-?dup in the proband. Real-time quantitative PCR confirmed that this 0.402 Mbp duplication involved part of intron 1 and exon 2, with a breakpoint within intron 2 (Fig. 1C). This duplication is not predicted to introduce a frameshift in the CASPR2 protein. The duplication was not inherited by the sibling.

PCR amplification and Sanger sequencing was performed to screen *CNTNAP2* exons in the family, revealing a paternally inherited missense variant c.2752C > T, p.(Leu918Phe) in the proband (Fig. 1B). This variant is absent from the gnomAD dataset (https://gnomad.broadinstitute.org/variant/) and affects a conserved residue within the Laminin G-like (LG) 3 domain (GERP = 5.49). It is reported In ClinVar (allele ID 924741, https://www.ncbi.nlm.nih.gov/clinvar/variation/949154/) and predicted damaging by in silico tools (e.g., DANN, 0.9977; Mutation Taster, 1; CADD, 24.1). Both variants were absent in the proband’s sibling. Further details are available in Supplementary Methods.

## Discussion

The range of clinical symptoms found in patients harboring biallelic *CNTNAP2* variants are collectively described as CASPR2 deficiency disorder. Dyskinetic features are not included amongst the classic neurological manifestations of this condition [1–4]. In this patient, we observed hyperkinetic stereotyped movements consisting of continuous, repetitive and rhythmic shaking of the four limbs, which became evident during the first months of life and persisted over the years. These abnormal movements did not resemble generalised dystonia, paroxysmal non-kinesigenic dyskinesia, or paroxysmal kinesigenic dyskinesia, but rather represent a novel neurological manifestation of CASPR2 deficiency disorder. This dyskinetic phenotype may be related to the role of CASPR2 in facilitating nerve conduction and synaptic connectivity, particularly given its widespread expression in the central and peripheral nervous system [5, 9, 14]. In patients harboring biallelic variants in *CNTNAP2*, brain MRI may be normal, such as in our case, or show cortical dysplasia (cortical dysplasia-focal epilepsy syndrome, CDFES).^1–3^ Additional findings include cerebellar abnormalities (vermian hypoplasia or atrophy) and nonspecific white matter abnormalities.^1,2^

The c.97 +?_209-?dup and p.(Leu918Phe) variants were inherited from unaffected parents, supporting the incomplete penetrance of heterozygous *CNTNAP2* variants [5, 6]. The maternally inherited variant (c.97 +?_209-?dup) involved a complete duplication of exon 2 (99 bp) (Fig. 1D), which may negatively impact protein folding and/or protein-protein interactions. Indeed, exon 2 encodes part of the discoidin homology domain, which is found in the extracellular portion CASPR2 and is known to mediate protein-protein interactions, pointing to a potential perturbation of this mechanism in the patient (Fig. 1E). The paternally inherited missense variant p.(Leu918Phe) is also in the extracellular portion of CASPR2, but is found in the third LG domain (Fig. 1E). Missense variants in the extracellular portion of CASPR2 have been shown to impair interactions with contactin 2 and affect axon growth in cortical neurons [12]. This effect is likely related to the trans-synaptic bridge formed in neurons by the interaction of CASPR2 and contactin 2 that contributes to synaptic organization and synaptic transmission [16]. It would therefore be of value to determine if the variants identified in this patient have functional consequences for protein-protein interactions, or for synaptic or juxtaparanodal organisation and signalling. Such functional studies may shed light on why heterozygous *CNTNAP2* variants show incomplete penetrance and why biallelic mutations together produce a range of phenotypes, including the novel motor phenotype described herein.

The maternal duplication also has the potential to alter the regulation of *CNTNAP2*. The transcription factor FOXP2 targets a binding site in intron 1 of *CNTNAP2* and regulates its expression. Mutations in *FOXP2* are a monogenic cause of childhood apraxia of speech (SPCH1, OMIM #602081) [17] and *CNTNAP2* is functionally implicated in the aetiology of this condition as part of the downstream network of FOXP2 target genes [18]. This is supported by a genetic association of *CNTNAP2* variants with phonological memory performance in children with specific language impairment [18]. Duplication of the FOXP2 target site in intron 1 of the maternally inherited allele could alter FOXP2-mediated regulation of *CNTNAP2* and lead to aberrant CASPR2 levels, a hypothesis that needs functional testing in cell or animal models. In this way, it is possible that the intronic duplication could contribute to neurodevelopmental or speech phenotypes in patients that are related to both FOXP2 and CNTNAP2, such as phonological processing.

This study has two main limitations. First, a proper functional characterization of the two detected *CNTNAP2* variants to evaluate their impact on protein structure and function could not performed. Second, it was not possible to further investigate the presence of additional variants with a potential modifier effect on the clinical phenotype through Next Generation Sequencing (NGS) techniques, such as whole exome sequencing (WES) or whole genome sequencing (WGS).

## Conclusion

This case of a boy with hyperkinetic stereotyped movements and biallelic *CNTNAP2* mutations expands our knowledge about *CNTNAP2*-related disorders. It presents a new, rare neurological manifestation for CDD and posits a remarkable molecular mechanism in which coding and non-coding *CNTNAP2* mutations could contribute to the observed phenotypes. Accordingly, we suggest screening *CNTNAP2* regulatory regions in patients with a CDD-suggestive phenotype even if a single heterozygous *CNTNAP2* variant has been identified or if atypical neurological phenotypes are also present. This will lead to better diagnoses that can improve the management of patients with these disorders. This, together with functional studies of the consequences of the identified mutations, will advance our scientific understanding of disease genes like *CNTNAP2*.

## Supplementary Information

**Additional file 3.**

**Additional file 4.**

## Data Availability

All data generated or analysed during this study are included in this published article.

## Abbreviations

ADHD: Attention deficit-hyperactivity disorder
ASD: Autism spectrum disorder
CDD: CASPR2 deficiency disorder
ID: Intellectual disability
NGS: Next Generation Sequencing
WES: Whole exome sequencing
WGS: Whole genome sequencing
