# Supplementary material for: Hyperkinetic stereotyped movements in a boy with biallelic CNTNAP2 variants
Source: Ital J Pediatr. 2021 Oct 12;47:208. doi: 10.1186/s13052-021-01162-w (PMC8507175; doi:10.1186/s13052-021-01162-w)
Supplement: Supplementary file 4 — Additional file 4. [file 13052_2021_1162_MOESM4_ESM.docx]

**Supplementary Material**

**1. Supplementary Methods**

**2. Supplementary Results**

**3. Supplementary Videos**

**1. Supplementary Methods**

*Genetic study*

Whole genome array-CGH (aCGH) was performed on genomic DNA extracted from peripheral blood using the Agilent 180K platform (Agilent, Santa Clara, California, United States; <https://www.agilent.com>), with an average resolution of approx. 40kb under optimal conditions. A variation in the number of copies was defined by a shift from the normal value of at least three consecutive probes for deletion and five for amplifications. The data was analysed using the Genomic Workbench software (Agilent, Santa Clara, California, United States; <https://www.agilent.com>) with human genomic DNA of the same sex as control (Agilent, Santa Clara, California, United States; <https://www.agilent.com>). Deletions and/or amplifications reported in the Database of Genomic Variants (<http://projects.tcag.ca/variation/>) were excluded (list available on request).

In order to test involvement of coding sequences in the maternal duplication, relative copy numbers were determined by real-time quantitative PCR (RT-qPCR) using iQ SYBR Green SuperMix (BioRad, Hercules, California, United Sates; <https://www.bio-rad.com>). Primers were designed for intron 1 (positive control), exon 2, intron 2 (95bp downstream of exon 2) and intron 2 (60kb downstream of exon 2, reference). The efficiency of primers was tested via five-fold serial dilution of human genomic DNA (EMD Millipore, Merck KGaA, Darmstadt, Germany; <https://www.merckmillipore.com/IT/it?bd=1>). Melting curve analysis was performed to assess the specificity of the amplification. Data analysis was performed using CFX manager software (BioRad, Hercules, California, United Sates; <https://www.bio-rad.com>). Differential copy numbers were determined by transforming Cq values to fold gene expression while taking into account the primer efficiency and subsequently normalizing for the reference. The sequences, amplicon sizes and efficiencies for the primers used are listed in the table below:

| Target | Forward primer | Reverse primer | Amplicon size (bp) | Primer efficiency |
| --- | --- | --- | --- | --- |
| CNTNAP2 intron 1 | CTGTTTCTCTGCATGCTGAC | GTATCCATACGGTAATGACACG | 210 | 88% |
| CNTNAP2 exon 2 | CAGAATTGCCTAAATTCCTTTGC | CAGAGACAAGTGGCTCATCAC | 164 | 75% |
| CNTNAP2 intron 2 | GCAGACACCAGAAATCACTC | GACGTACTATCTCTAACTTCC | 125 | 88% |
| CNTNAP2 intron 2 (reference) | CCCACATAGAATGGACACGTAGAA | CCTCCCCCTGTGTATTTGCG | 100 | 98% |

All exons of *CNTNAP2* were screened using PCR and Sanger sequencing. PCR amplification was performed using Taq DNA polymerase kit (Invitrogen, Waltham, Massachusetts, United States; <https://www.thermofisher.com/it/en/home/life-science/pcr/pcr-enzymes-master-mixes/invitrogen-taq-dna-polymerases.html>) as per supplier’s instructions. PCR products were purified from gel with the Wizard SV gel clean-up kit (Promega, Milan, Italy; <https://ita.promega.com/products/nucleic-acid-extraction/clean-up-and-concentration/wizard-sv-gel-and-pcr-clean-up-system/?catNum=A9281>) and Sanger sequenced to identify CNTNAP2 mutations. The following primers were used for amplification and sequencing:

| Target | Forward primer | Reverse primer | Amplicon size (bp) |
| --- | --- | --- | --- |
| CNTNAP2 exon 1 | CAGCCCATCTCCCTTCAAGA | GCTGGGTTTCGAGTTTGTCT | 353 |
| CNTNAP2 exon 2 | GGAGTCCTCTTTGTCTTTCCTCC | GAGTGATTTCTGGTGTCTGCC | 630 |
| CNTNAP2 exon 3 | GCACTGCCAAGACCAATTAAGA | TCTTTGCTTTCCTGCCAATGA | 322 |
| CNTNAP2 exon 4 | TCACAAGCCCTACCATTGGA | ACATGCAAATAGAGACACAACCT | 442 |
| CNTNAP2 exon 5 | AGAGGACTGTCAATTTCTCAAGA | ACGGAAAGAATAGAACTGACAGT | 362 |
| CNTNAP2 exon 6 | TCCCAGGTTAACTCGAATGGA | GCCTGGATAGCATGGTTCCT | 573 |
| CNTNAP2 exon 7 | TGCCATAGATTTTGGAGGCA | TGCGGGTGAAAATCCTTACC | 307 |
| CNTNAP2 exon 8 | AGGCTGTGCTTCAAAACTTGT | ACCTAATCCTGAGCGTGTAACA | 474 |
| CNTNAP2 exon 9 | GGAAATTGTGTTCAGCTGGGT | TGCTCAGTGGAATTACAGTTTGT | 352 |
| CNTNAP2 exon 10 | TGAGTGACAGTAGACCCCAG | TTGGACAACGTGAGGTAGGA | 457 |
| CNTNAP2 exon 11 | CGCTTGGCACCTTCTTTCAT | TGTGGATTATTCAGGTTGCTGA | 377 |
| CNTNAP2 exon 12 | TCTGGGGAGCCATTTGTTCT | TGGGCTGAACTTTGCAACTT | 467 |
| CNTNAP2 exon 13 | CTGTTCTACACCAGCTCAGTAA | CCCAGAAAACAAGCCCAATGAT | 478 |
| CNTNAP2 exon 14 | GGGTGTAAGTGTGGCAGTCT | TCTAAATGCATACCCTTGTCGC | 436 |
| CNTNAP2 exon 15 | TGTCTAATGCAGCCTCCTCA | AATCTCGGCTCCTGTACTGG | 365 |
| CNTNAP2 exon 16 | CATGACTAGGCTGATCAGGGT | TGCTTCCCTGAGAGCATCC | 426 |
| CNTNAP2 exon 17 | CCATTGATTTTGCCATCGACC | GGCCAACACCTTTACTTTTGG | 494 |
| CNTNAP2 exon 18 | GCTATGCAGTGTCATCTCCT | TCCACCTTACCTTTGTTGCA | 348 |
| CNTNAP2 exon 19 | GGTATCGGCATCAGACCTCT | GTCTGCCCAAATGTTAAATGGC | 339 |
| CNTNAP2 exon 20 | AGCAGGAATTGAGGGGATGT | CCCTCAAAACAAAACCAATGGC | 301 |
| CNTNAP2 exon 21 | ACAGGGTAGAGACGTGCTTC | TGCCCAGCCTAATCACAATG | 398 |
| CNTNAP2 exon 22 | ACAAGCATTCAAAGACAGGTATG | TCCATTCCATAGTCCCAAGAGA | 393 |
| CNTNAP2 exon 23 | CTCGTCTGTCTGTGGAACTAGA | TCCATAGTTGAGTAGCCCCA | 423 |
| CNTNAP2 exon 24 | GTGTCTGACGGAGCTGTAGT | TCCTCCCTATCCCATAGCCA | 317 |

**2. Supplementary Results**

The variant c.2752C>T, p.(Leu918Phe) has been submitted to the LOVD database (<https://www.lovd.nl>): variant ID #0000709038 (NC_000007.13:chr7-147844780-C-T, CNTNAP2(NM_014141.5):c.2752C>T). The variant c.97+?_209-?dup has been submitted to the LOVD database (<https://www.lovd.nl>): Variant ID #0000709039 (NC_000007.13:?, CNTNAP2(NM_014141.5):c.97+?_209-?dup)

**3. Supplementary Videos**

**Supplementary video 1** (See separate file). This video shows the patient at 3 months of age. Videos were taken in a home environment while the child was playing with his parents. Hyperkinetic stereotyped movements can be observed at 11-16 sec., 19-26 sec, 30-38 sec.

**Supplementary video 2** (See separate file). This video shows the patient at 6 years of age. Videos were taken in a home environment while the child was seated on mother’s knees. Non-triggered fast, high-amplitude, rhythmic, continuous, and repetitive shaking involving the four limbs with stereotypic features can be observed during the whole video.

**Supplementary video 3** (See separate file). This video shows the patient at 6 years of age. Videos were taken in a home environment while the child is eating, next to the unaffected sibling. Non-triggered fast, high-amplitude, rhythmic, continuous, and repetitive shaking involving the four limbs with stereotypic features can be observed at 13-20 sec., 30-42 sec., 50 sec.-1:06 min., 1:20-1:50 min.
